# Supplementary material for: Transcriptional response to hepatitis C virus infection and interferon‐alpha treatment in the human liver
Source: EMBO Mol Med. 2017 Mar 30;9(6):816–34. doi: 10.15252/emmm.201607006 (PMC5452008; doi:10.15252/emmm.201607006)

Table of Contents

Appendix Figure Legends . . . 1
Appendix Figures
Appendix Figure S1 . . . 5
Appendix Figure S2 . . . 6
Appendix Figure S3 . . . 7
Appendix Figure S4 . . . 8
Appendix Figure S5 . . . 9
Appendix Figure S6 . . . 10
Appendix Figure S7 . . . 11
Appendix Figure S8 . . . 12
Appendix Figure S9 . . . 13
Appendix Figure S10 . . . 14
Appendix Figure S11 . . . 15
Appendix Figure S12 . . . 16

Appendix Figure Legends

Appendix Figure S1.

A. Principal component analysis (PCA) of protein-coding gene expression levels, for untreated CHC and control samples. The log2-transformed RPKM levels of all expressed protein-coding genes were used as input for the PCA (Materials and Methods). Black: non-CHC; green: CHC low ISG; red: CHC high ISG samples.

B. Same as **A**, for protein-coding genes associated with "response to interferon" Gene Ontology terms (Materials and Methods).

Appendix Figure S2.

A. Results of the Homer transcription factor binding enrichment analysis (Materials and Methods), for protein-coding genes that are up-regulated in CHC low ISG compared to control non-CHC samples. The numbers in parentheses represent the percentage of genes that have this motif in the promoter region and the estimated enrichment with respect to the genomic background.

B. Boxplots of the intensity of the expression change (log2 fold) between non-CHC patients and CHC low ISG patients, for different categories of protein-coding genes defined based on the miRNAs that are predicted to target them. Target predictions were determined with HITS-CLIP in Huh7 cells (Luna et al, 2015). From left to right: genes that are not targets of any expressed miRNA; genes that are targeted by at least one expressed miRNA; genes that are targeted by miR-122-5p, miR-192/215, let7a-i/miR-98 and miR-15, respectively.

C. Boxplots of the intensity of the expression change (log2 fold) between non-CHC patients and CHC low ISG patients, for different categories of protein-coding genes defined based on the number of miRNA families that are predicted to target them. Only miRNAs with high expression in normal or HCV-infected liver were analyzed (Hou et al, 2011). Evolutionarily conserved miRNA target predictions were extracted from TargetScan v7.1 (Agarwal et al, 2015) (Materials and Methods).

Appendix Figure S3.

A. Results of the Homer transcription factor binding enrichment analysis (Materials and Methods), for protein-coding genes that are up-regulated in CHC high ISG compared to control non-CHC samples. The numbers in parentheses represent the percentage of genes that have this motif in the promoter region and the estimated enrichment with respect to the genomic background.

B. Same as **A**, for genes down-regulated in CHC high ISG compared to control non-CHC samples.

C. Barplots representing the expression patterns of three categories of genes (all expressed genes, genes up-regulated in CHC high ISG patients, and genes down-regulated in CHC high ISG patients compared to control non-CHC samples), in the GTEx tissue transcriptome collection. For each expressed gene, we scored the tissue or cell type in which its maximum expression level was reached. The height of the rectangles represents the percentage of genes that reaches maximum expression in each of the tissues.

Appendix Figure S4.

A. Volcano plot for the differential expression analysis between CHC patients with low levels of endogenous ISG activation (CHC low ISG) and CHC patients with high levels of endogenous ISG activation (CHC high ISG). The X-axis represents the log2 fold expression change in CHC high ISG compared to CHC low patients. The Y-axis represents the false discovery rate (with a -log10 transformation) of the differential expression test. Protein-coding genes, candidate long non-coding RNAs (lncRNAs) and other gene categories (including pseudogenes and transcripts with unclear coding potential, Materials and Methods) are represented separately. Only genes with false discovery rate (FDR) $< 10\%$ and with

fold expression change > 1.5 are shown.
B. Heatmap of the expression patterns of the top differentially expressed genes between CHC low ISG and CHC high ISG patients. To select the top differentially expressed genes, we set a minimum absolute fold change threshold of 3.5 for up-regulated genes and of 2 for down-regulated genes. The heatmap represents the Z-score of the log2-transformed RPKM (reads per kilobase of exon per million mapped reads) gene expression levels, normalized based on housekeeping genes (Materials and Methods).
C. Results of the Homer transcription factor binding enrichment analysis (Materials and Methods), for protein-coding genes that are up-regulated in CHC high ISG compared to CHC low ISG samples. The numbers in parentheses represent the percentage of genes that have this motif in the promoter region and the estimated enrichment with respect to the genomic background.

Appendix Figure S5.
A. Results of the Homer transcription factor binding enrichment analysis (Materials and Methods), for protein-coding genes that are up-regulated following 4h of pegIFNa/ribavirin treatment. The numbers in parentheses represent the percentage of genes that have this motif in the promoter region and the estimated enrichment with respect to the genomic background.
B. Same as **A**, for genes that are up-regulated following 16h of pegIFNa/ribavirin treatment.
C. Same as **A**, for genes that are up-regulated following 48h of pegIFNa/ribavirin treatment.
D. Same as **A**, for genes that are up-regulated following 96h of pegIFNa/ribavirin treatment.
E. Same as **A**, for genes that are up-regulated following 144h of pegIFNa/ribavirin treatment.
F. RNA-seq profiles along the NRIR lncRNA, for pre-treatment and post-treatment biopsies at the 4h time point. Gray: normalized read coverage for baseline/pre-treatment biopsies. Black: normalized read coverage for post-treatment biopsies. Red: positive difference between post-treatment and pre-treatment biopsies. Green: negative difference between post-treatment and pre-treatment biopsies.
G. Same as **F**, for the lncRNA CTD-2521M24.5.
H. Same as **F**, for the lncRNA CTD-2095E4.5.

Appendix Figure S6.
A. Differential expression patterns of the genes that are significantly up-regulated (FDR < 0.1 and minimum absolute fold change 1.5) in CHC high ISG patients compared to CHC low ISG patients. Blue dots: genes also significantly differentially expressed following pegIFNa/ribavirin treatment (FDR <0.05); red dots: genes significant only in the comparison between low ISG and high ISG samples; black dots: genes not tested in the pegIFNa/ribavirin comparison due to low or highly variable expression levels (Materials and Methods). X-axis: log2 fold expression change in pegIFNa-treated compared to control biopsies. Y-axis: log2 fold expression change in high ISG patients compared to low ISG patients. The numbers of the genes in each category are depicted on the plot area, with the same color code.
B. Similar to **A**, for genes that are significantly down-regulated (FDR < 0.1 and minimum absolute fold change 1.5) in CHC low ISG and CHC high ISG patients.
C. Histogram of the difference in expression levels (log2-transformed RPKM) between samples treated with pegIFNa/ribavirin and CHC high ISG samples, for the genes that are up-regulated (FDR < 0.1 and minimum absolute fold change 1.5) in CHC high ISG patients compared to CHC low ISG patients. The differences were computed between expression levels averaged across all relevant samples.
D. Similar to **C**, for genes that are significantly down-regulated (FDR < 0.1 and minimum absolute fold change 1.5) in CHC high ISG patients compared to CHC low ISG patients.

Appendix Figure S7.
A. Principal component analysis (PCA) of protein-coding gene expression levels, for treated and untreated CHC. The log2-transformed RPKM levels of protein-coding genes that are differentially expressed following pegIFNa treatment (minimum absolute fold change 2, FDR<0.05, at any of the 5 time points) were used as input for the PCA. Green: CHC low ISG, before treatment; orange: CHC high ISG samples, before treatment; red: pegIFNa/ribavirin-treated samples. The dot shapes depict the time points of the pegIFNa treatment.
B. Hierarchical clustering analysis of treated and untreated CHC samples. For the hierarchical clustering, pairwise distances between samples were computed as 1-Pearson's correlation coefficient, using protein-coding genes that are differentially expressed following pegIFNa treatment (minimum absolute fold change 2, FDR<0.05, at any of the 5 time points). The heatmap represents pairwise distances between samples. Cold colors (blue) represent small distances; warm colors (yellow) represent large distances.

Appendix Figure S8.
Heatmap of the expression patterns of 100 selected protein-coding genes, in control non-CHC samples, CHC low ISG and high ISG samples (pre-treatment) and pegIFNa/ribavirin-treated samples. The selected genes were strongly differentially expressed (minimum absolute fold change 2, FDR<0.01) following pegIFNa/ribavirin treatment for at least 2 time points, but were not significantly differentially expressed between low ISG and high ISG samples (FDR >= 0.1). With these settings, 85 up-regulated and 25 down-regulated genes were included. The heatmap represents the Z-score of the log2-transformed RPKM expression levels. Cold colors represent low expression values; warm colors represent high expression values.

Appendix Figure S9.
A. RNA-seq profiles along the miR-331/miR-3685 host gene, for pre-treatment and post-treatment biopsies at the 16h time point, for the three analyzed individuals (I1, I2 and I3). Gray: normalized read coverage for baseline/pre-treatment biopsies. Black: normalized read coverage for post-treatment biopsies. Red: positive difference between post-treatment and pre-treatment biopsies. Green: negative difference between post-treatment and pre-treatment biopsies.
B. Same as **A**, for the miR-192/miR-194 host gene.
C. Same as **A**, for the miR-146a host gene.

Appendix Figure S10.
Quantification of mature miRNA levels by qPCR (Materials and methods). We note that qPCR could not be performed for some samples due to low remaining RNA quantity (Dataset EV 12).
A. Mean Ct value (average of 3 technical replicates, normalized by total RNA quantity) for miR-122-5p, before and after pegIFNa/ribavirin treatment, at the 5 time points. Black dots: before treatment; red dots: after treatment. Data from paired biopsies are connected with a dotted line.
B. Log2 fold change (difference between Ct values before and after treatment) for miR-122-5p. The dotted red line represents a null fold change. Each dot represents a pair of pre-treatment and post-treatment biopsies.
C. Same as **A**, for miR-146a-5p.
D. Same as **B**, for miR-146a-5p.
E. Same as **A**, for miR-331-3p.
F. Same as **B**, for miR-331-3p.

Appendix Figure S11.

A. Dot-chart of the median fold expression change following pegIFNa/ribavirin treatment at the 4h time point, for predicted targets of miRNAs whose hosts are down-regulated (red dots) or of other miRNAs (black dots). We show 79 miRNA families with at least 100 conserved target genes. As a control, we show the median fold expression change for all genes predicted to be targets of any liver-expressed miRNAs or of genes not predicted to be targeted by these miRNAs (blue dots). The horizontal bars represent 95% confidence intervals of the median, constructed with a bootstrap resampling approach (Materials and Methods). Only miRNAs with high expression in normal or HCV-infected liver were analyzed (Hou et al, 2011). Evolutionarily conserved miRNA target predictions were extracted from TargetScan v7.1 (Agarwal et al, 2015) (Materials and Methods). miRNAs highlighted in red correspond to primary miRNA transcripts significantly down-regulated at the 4h time point; miRNAs highlighted in orange correspond to primary miRNA transcripts significantly down-regulated at other time points.
B. Same as **A**, for the 48h time point.

Appendix Figure S12.

A. Dot-chart of the median fold expression change following pegIFNa/ribavirin treatment at the 96h time point, for predicted targets of miRNAs whose hosts are down-regulated (red dots) or of other miRNAs (black dots). We show 79 miRNA families with at least 100 conserved target genes. As a control, we show the median fold expression change for all genes predicted to be targets of any liver-expressed miRNAs or of genes not predicted to be targeted by these miRNAs (blue dots). The horizontal bars represent 95% confidence intervals of the median, constructed with a bootstrap resampling approach (Materials and Methods). Only miRNAs with high expression in normal or HCV-infected liver were analyzed (Hou et al, 2011). Evolutionarily conserved miRNA target predictions were extracted from TargetScan v7.1 (Agarwal et al, 2015) (Materials and Methods). miRNAs highlighted in red correspond to primary miRNA transcripts significantly down-regulated at the 96h time point; miRNAs highlighted in orange correspond to primary miRNA transcripts significantly down-regulated at other time points.
B. Same as **A**, for the 144h time point.

Appendix Figure S1

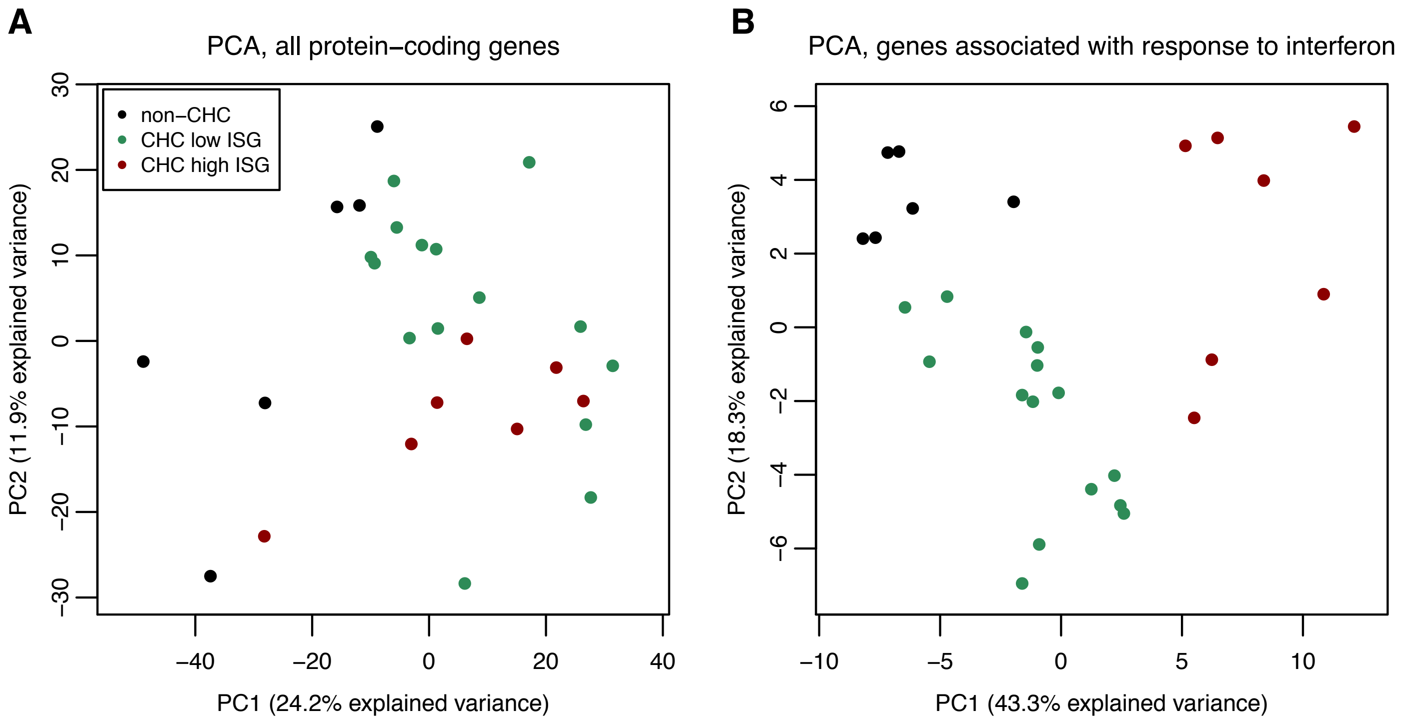

Appendix Figure S2

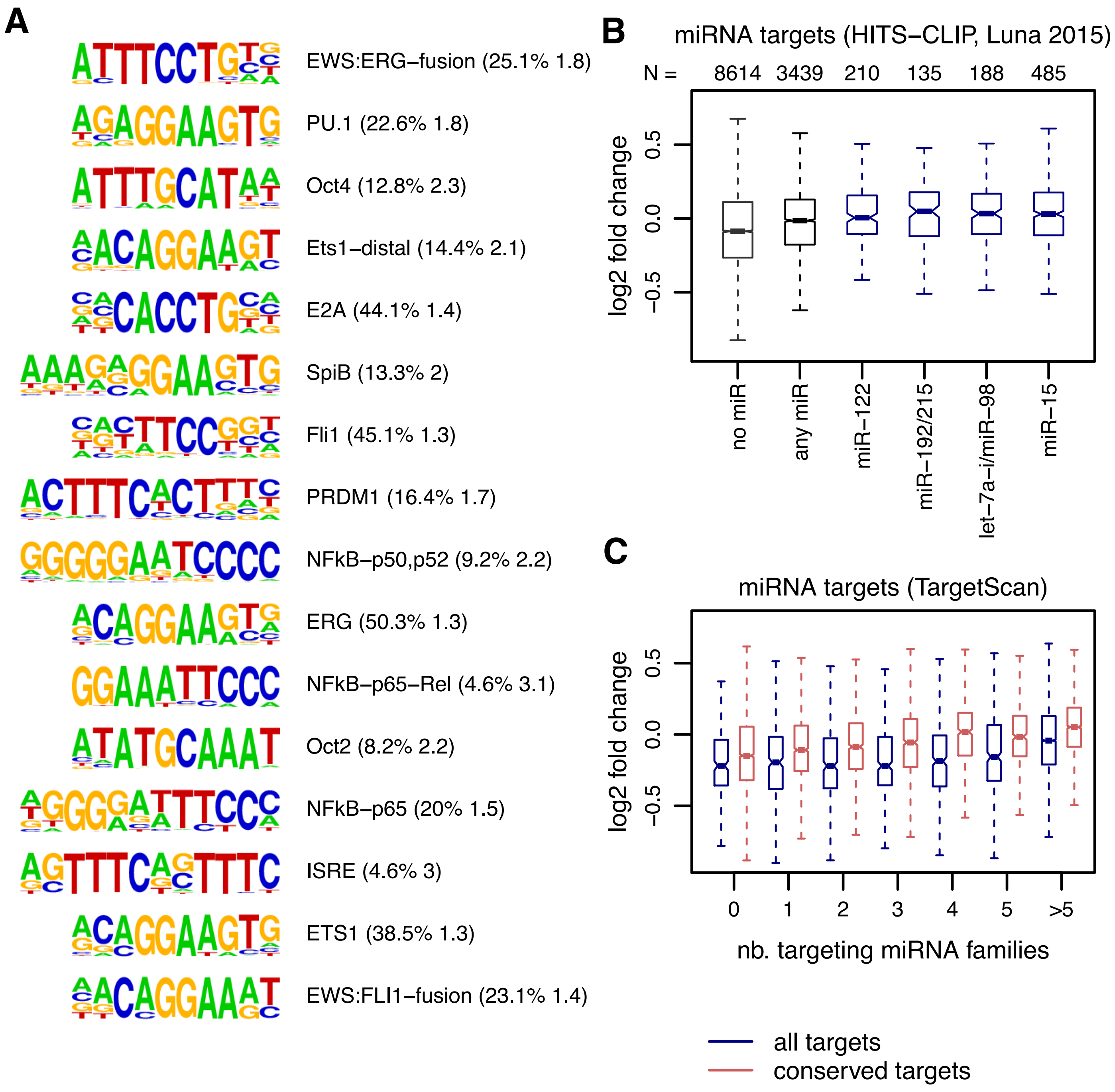

Appendix Figure S3

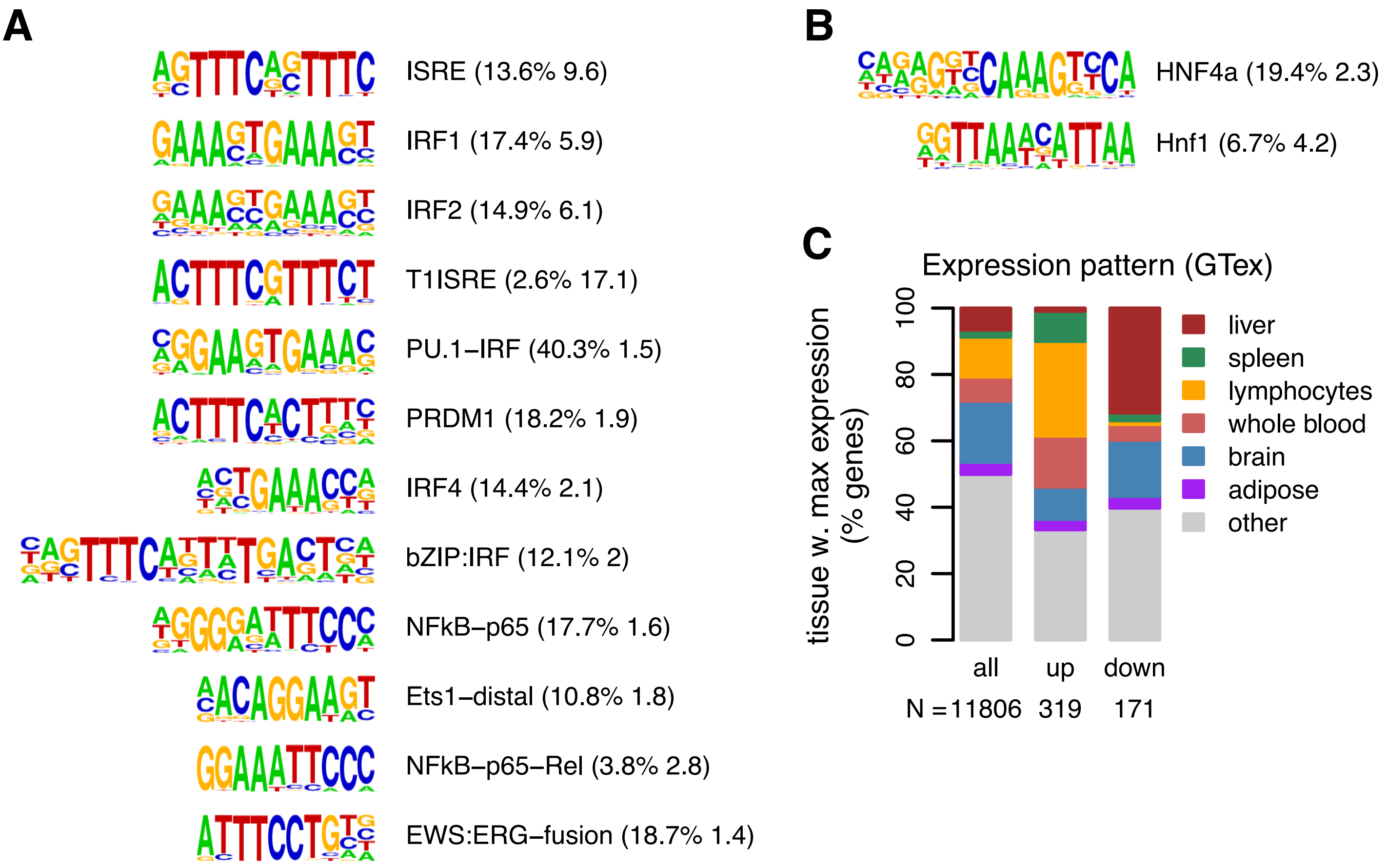

Appendix Figure S4

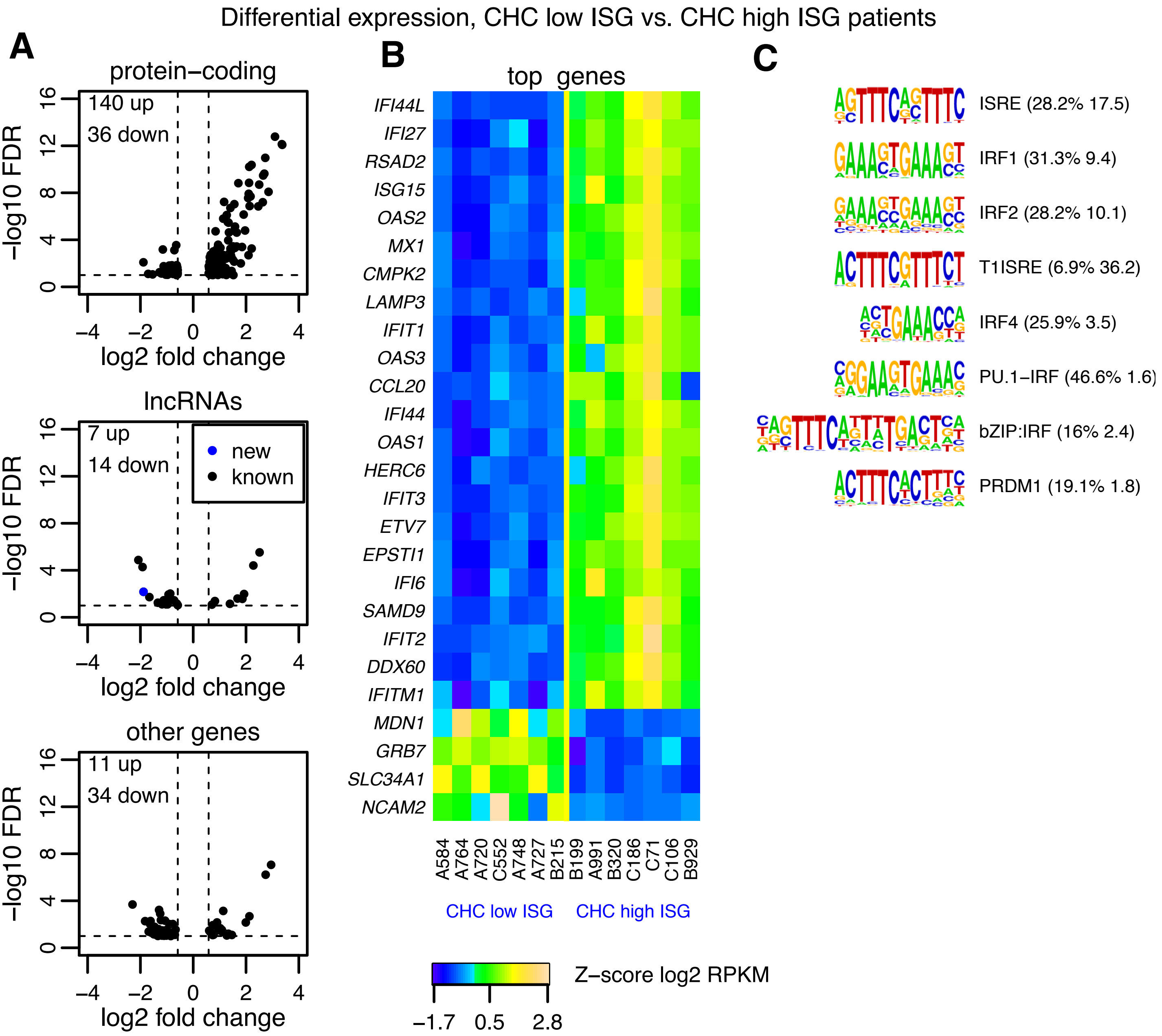

Appendix Figure S5

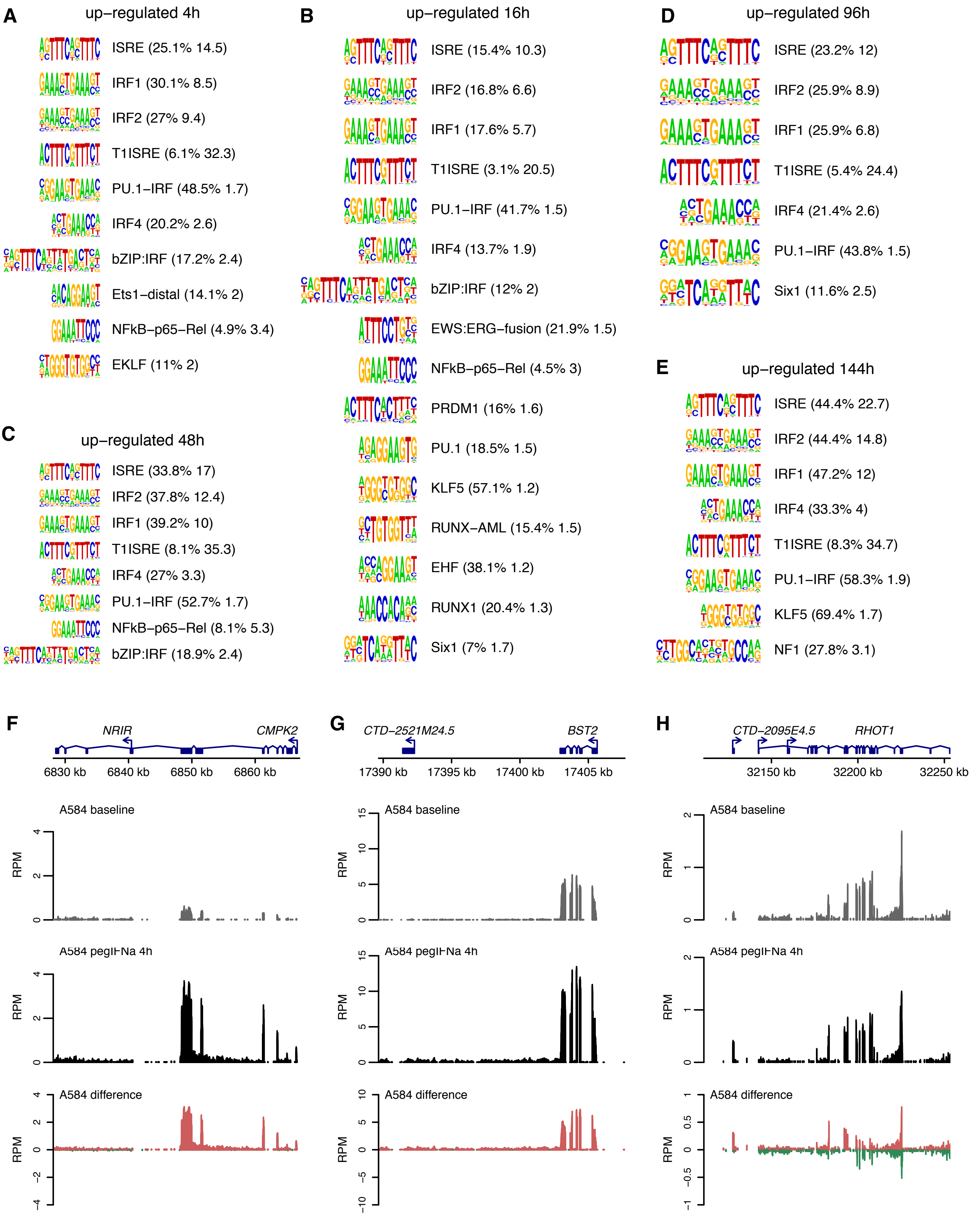

Appendix Figure S6

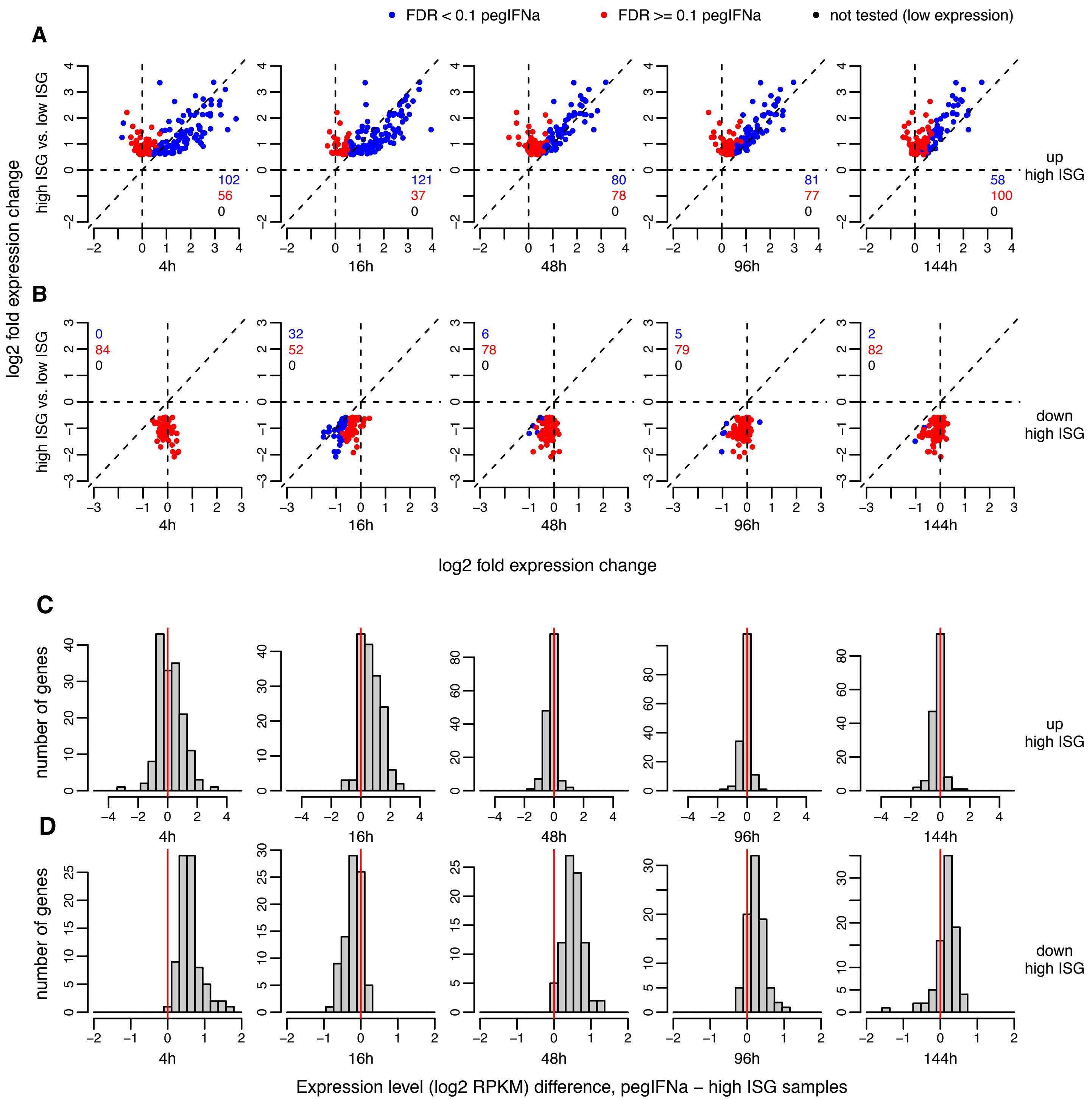

Appendix Figure S7

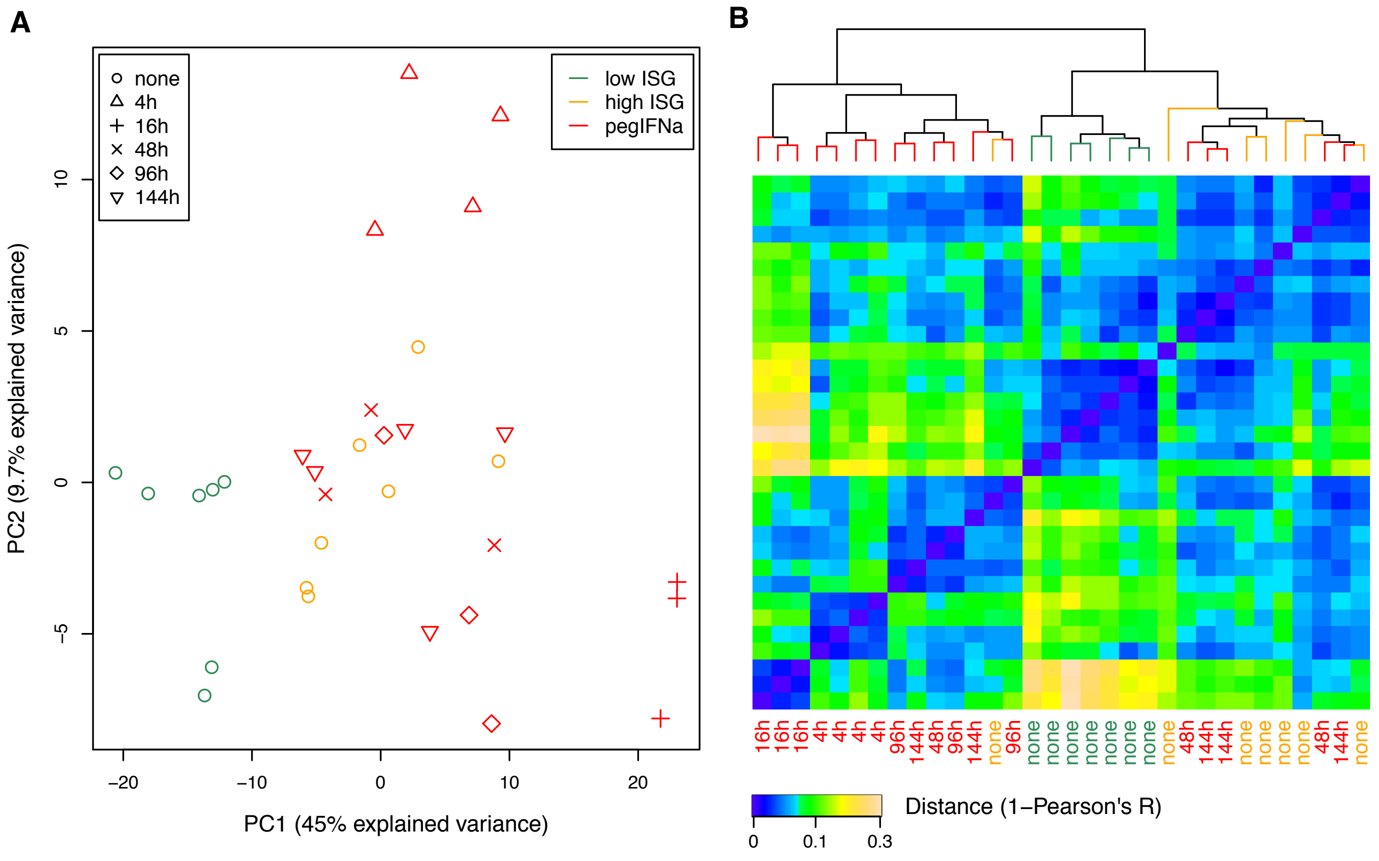

Appendix Figure S8

differentially expressed after pegIFNa, no difference between low ISG and high ISG patients

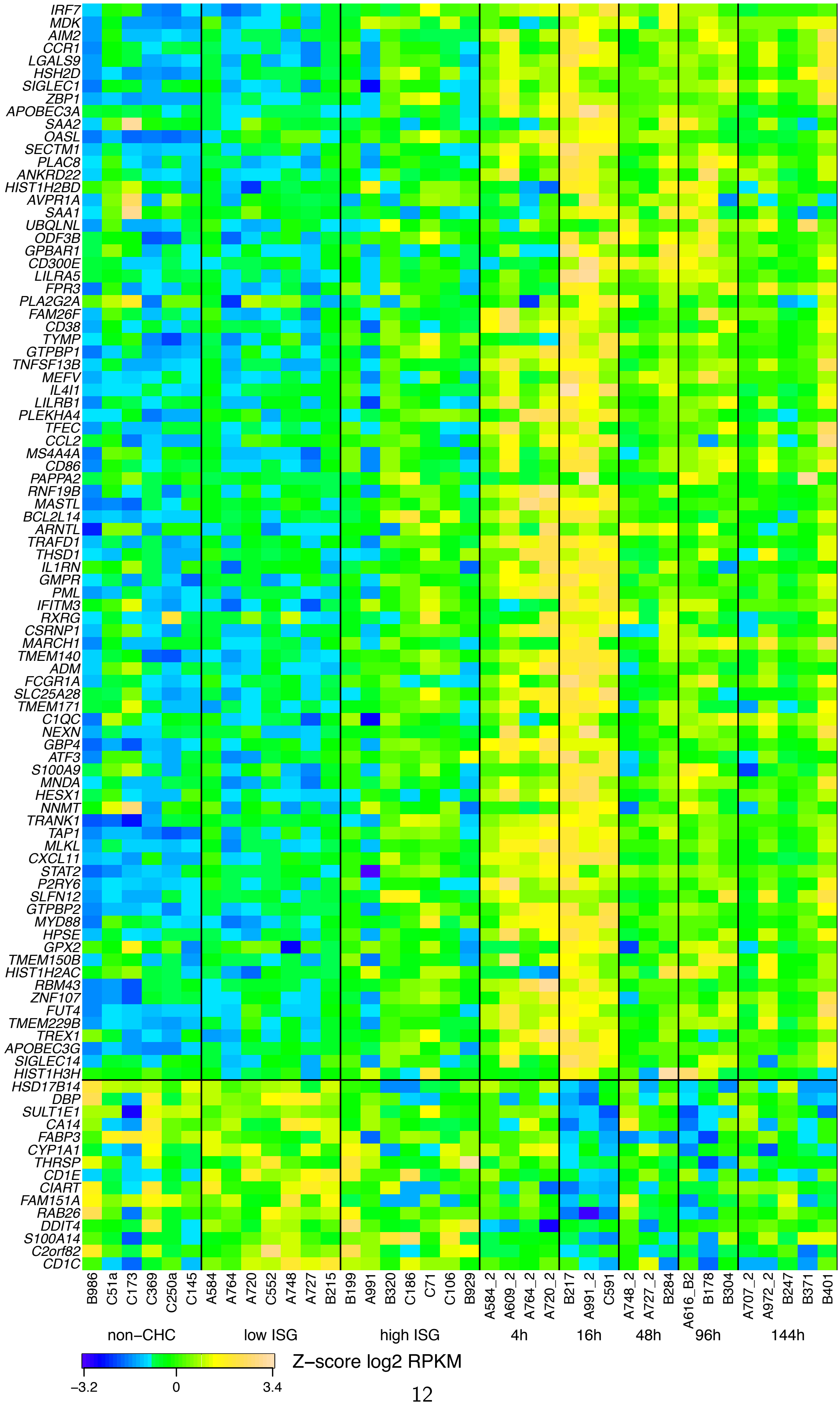

Appendix Figure S9

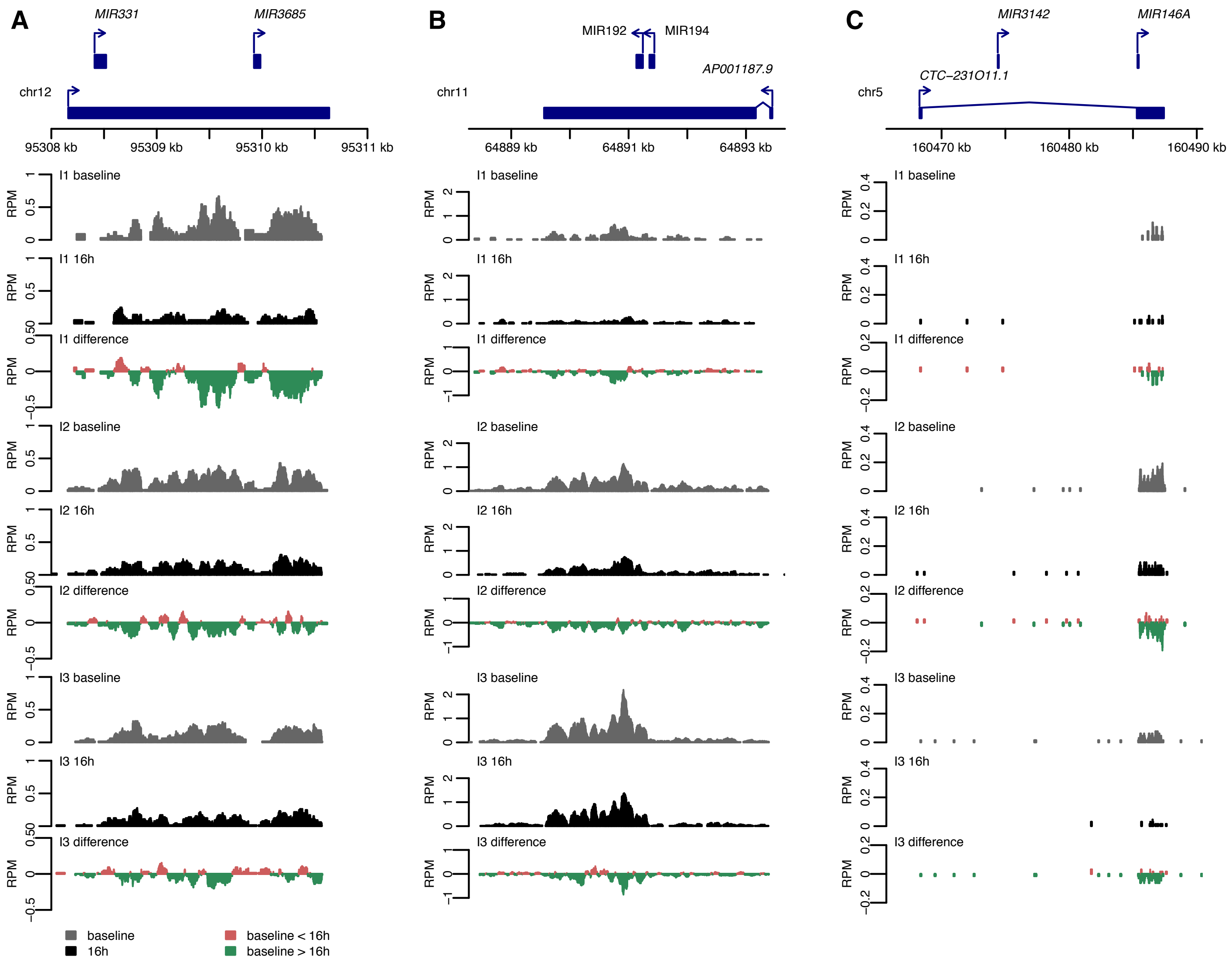

Appendix Figure S10

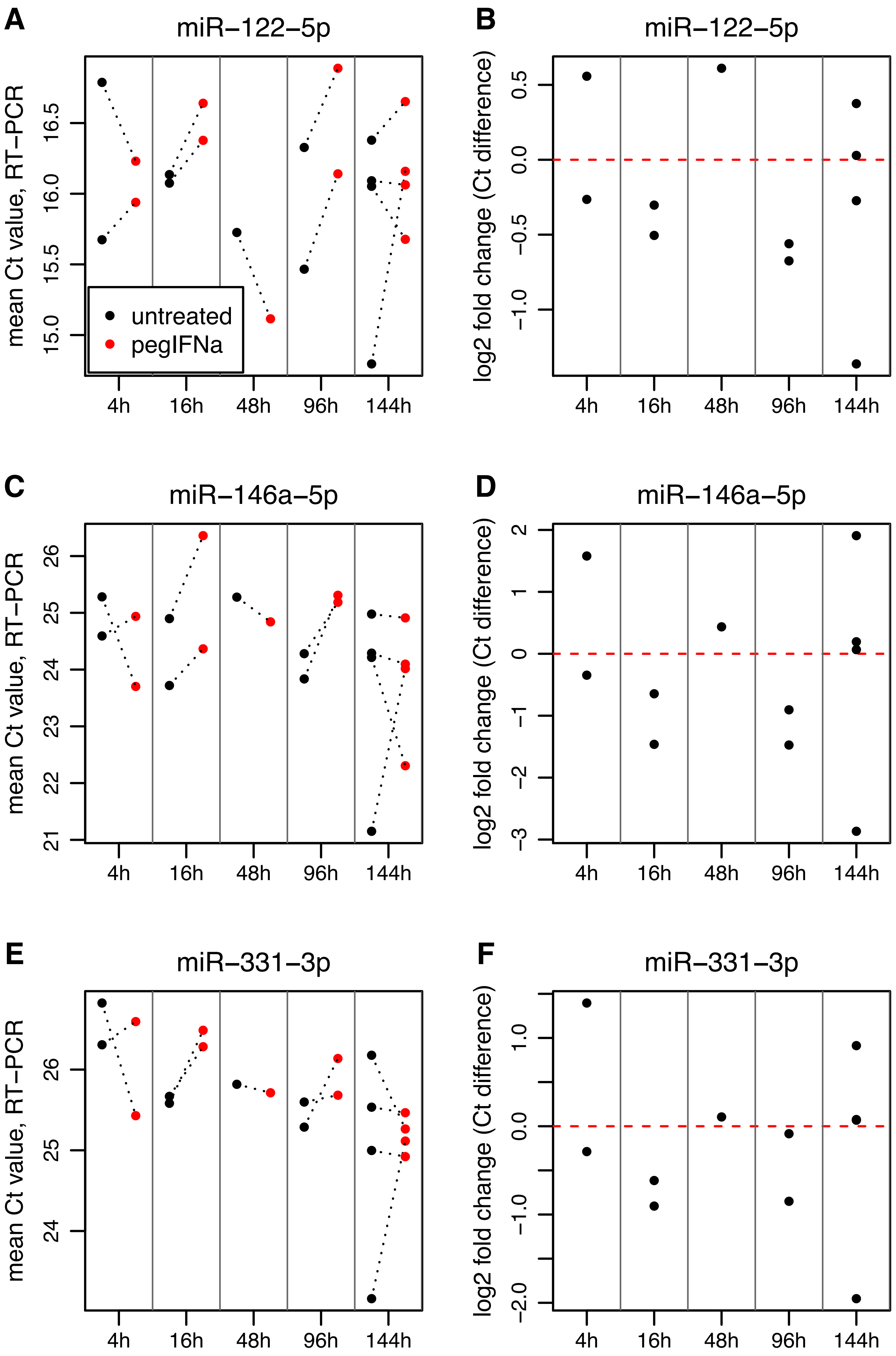

Appendix Figure S11

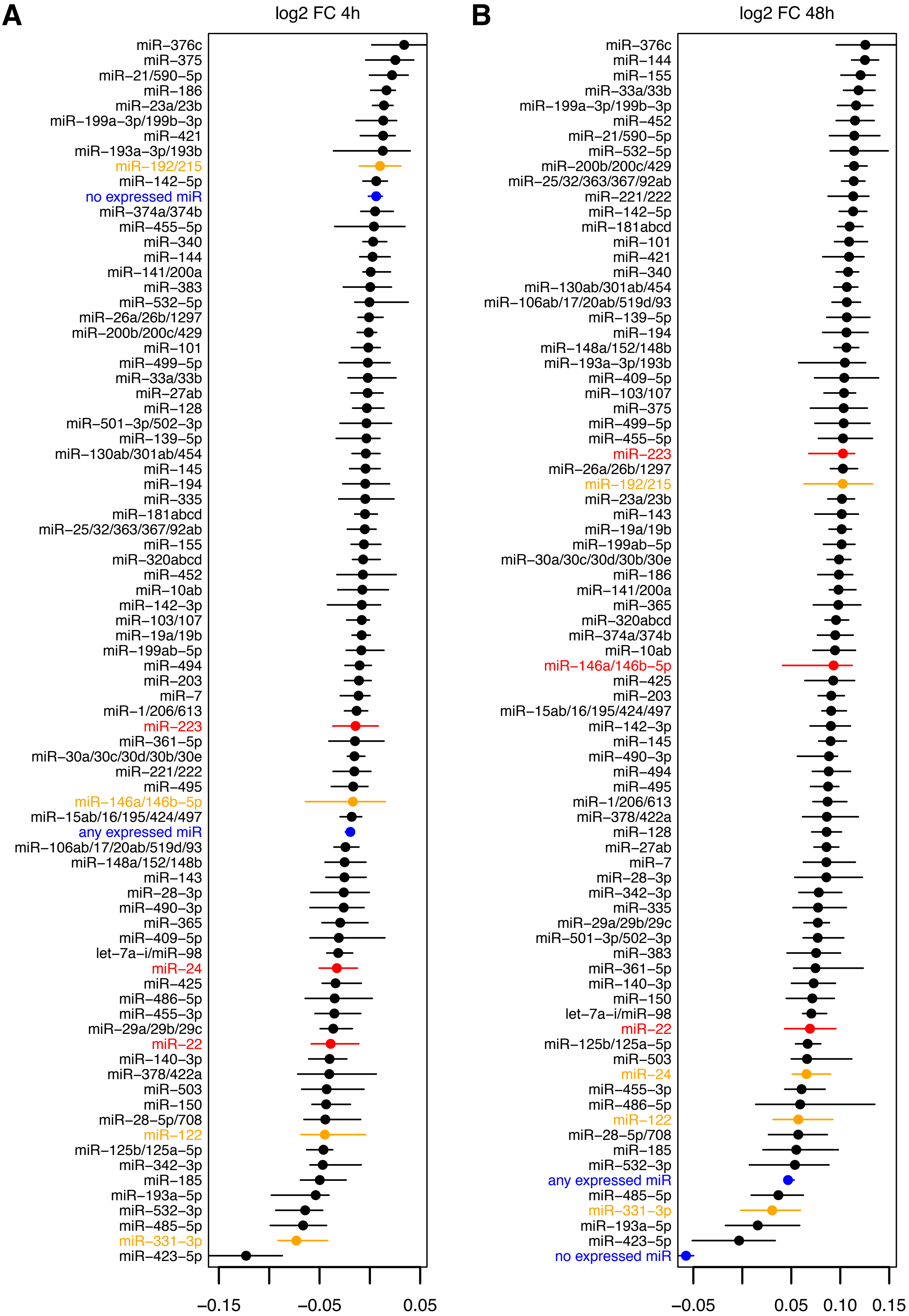

Appendix Figure S12

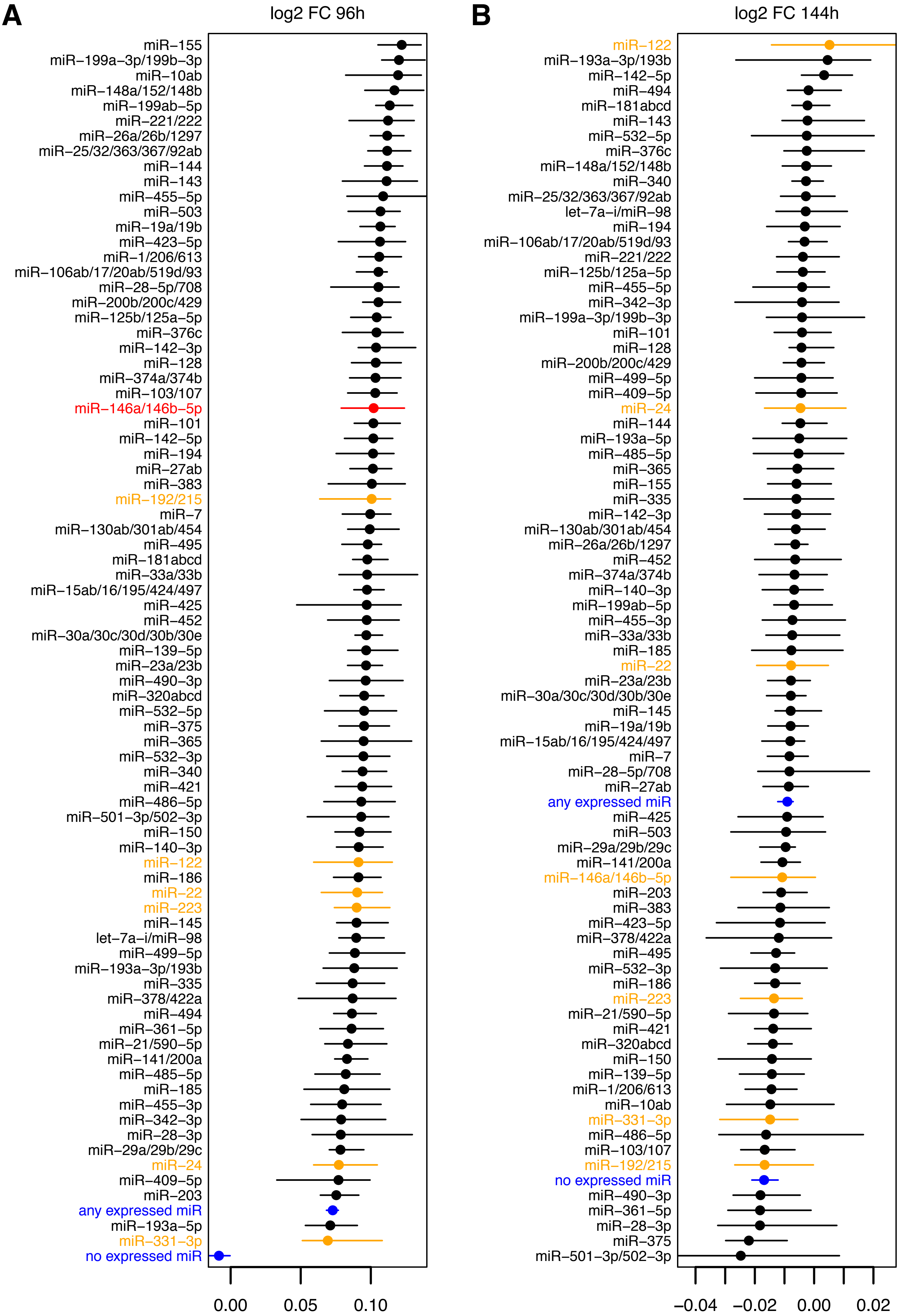

Supplement: Supplementary file 1 — Appendix [file EMMM-9-816-s001.pdf]
